# The Role of IL-6 in the Diagnosis of Neonatal Sepsis and Its Influence on Maternal Mental Health

**DOI:** 10.7759/cureus.80693

**Published:** 2025-03-17

**Authors:** Simona I Sipos, Daliborca Cristina Vlad, Virgil R Enatescu, Radu D Moleriu, Ion Petre, Cristina G Balasan, Luciana Marc, Ramona E Dragomir, Corina Vernic

**Affiliations:** 1 Pharmacology, "Victor Babes" University of Medicine and Pharmacy, Timisoara, ROU; 2 Pharmacology, “Victor Babes” University of Medicine and Pharmacy, Timisoara, ROU; 3 Psychiatry, "Victor Babes" University of Medicine and Pharmacy, Timisoara, ROU; 4 Medical Informatics and Biostatistics, "Victor Babes" University of Medicine and Pharmacy, Timisoara, ROU; 5 Genetics, "Pius Branzeu" County Emergency Clinical Hospital, Timisoara, ROU; 6 Nephrology, "Victor Babes" University of Medicine and Pharmacy, Timisoara, ROU; 7 Obstetrics and Gynecology, "Alessandrescu-Rusescu" National Institute for Mother and Child Health, Bucharest, ROU

**Keywords:** crp: c-reactive protein, diagnosis of neonatal sepsis, interleukin-6 (il-6), maternal mental health, neonatal sepsis

## Abstract

Neonatal sepsis (NS) remains a significant cause of morbidity and mortality in newborns, particularly in preterm and low-birthweight infants. It has been shown previously that interleukin-6 (IL-6) can predict sepsis, and its non-invasive determination can be performed quickly, leading to the diagnosis and appropriate treatment for each case.

Elevated IL-6 levels have been associated with early and late-onset NS, showing potential as an early indicator of infection before the onset of clinical symptoms. IL-6, in conjunction with other biomarkers such as C-reactive protein (CRP) and procalcitonin, may improve the accuracy of sepsis diagnosis and help guide antibiotic therapy.

We conducted a retrospective study to assess the validity of IL-6 in predicting NS. The optimal IL-6 cutoff was 181 pg/ml, yielding a sensitivity of 80.1%, a specificity of 85.7%, a positive predictive value of 84.6%, and a negative predictive value of 81.8%. Among culture-positive cases, IL-6 sensitivity was 90%, while in culture-negative cases, sensitivity was 71.4%. Our study showed that IL-6 is a new biomarker with high sensitivity and good specificity for identifying sepsis, and it has been linked with a better diagnostic value than CRP.

## Introduction

Neonatal sepsis (NS) is a potentially life-threatening clinical condition that causes significant morbidity and mortality, especially in preterm newborns. Multiple studies have demonstrated that interleukin-6 (IL-6) could be an effective, non-invasive, and rapid tool for diagnosing NS [1-3]. NS continues to remain an important cause of morbidity and mortality among newborns, especially in countries with limited resources; therefore, healthcare systems must place a high priority on prevention, early detection, and efficient management techniques. Because NS has the potential to save lives and improve the health of affected infants, it is crucial to recognize and treat it [1-3].

NS can be classified as either early-onset or late-onset, depending on when it first manifests. Early-onset sepsis (EOS) refers to a bacterial infection occurring within the first 72 hours of life, typically caused by pathogens acquired during labor or delivery, while late-onset sepsis (LOS) occurs after the first 72 hours, usually between 4 and 28 days of life. Both types require prompt diagnosis and treatment to reduce the risk of complications.

The prognosis and outcome of NS depend on the speed with which the diagnosis is made and on-time and efficient antibiotic therapy. For a neonatologist, the diagnosis of NS can be one of the most difficult challenges. Among numerous biomarkers, IL-6 could be a fast and reliable predictor for early diagnosis of NS [4-11]. The primary effects of IL-6 on lymphoid and non-lymphoid cells involve regulating the body’s immune and inflammatory responses. While many of these functions match with those of type 1 interleukin (IL-1), including the synthesis of acute-phase reactants and the induction of fever, IL-6 also exhibits unique anti-inflammatory properties [12,13].

The objective of our study was to assess the diagnostic validity of IL-6 as a reliable biomarker for predicting NS, in order to enhance early detection and improve clinical outcomes.

## Materials and methods

We conducted a retrospective study to determine the validity of IL-6 in predicting NS for one year (March 2023 to March 2024), using previously collected data to investigate the factors associated with NS. Serum IL-6 levels were measured using the ELISA method in 41 neonates with suspected sepsis and 42 healthy neonates without clinical or laboratory evidence of infection.

For the statistical analysis, we gathered 200 patients treated in our clinic ("Pius Branzeu" County Emergency Clinical Hospital, Timisoara, Romania) for one year. Statistical power analysis was used to determine the sample, accounting for the expected prevalence of newborn sepsis, the desired confidence level, and the expected effect size. It was concluded that this sample size would offer adequate statistical power to identify significant differences and guarantee trustworthy findings for our study's objectives.

The data for this retrospective study were collected from medical records, which included both clinical information and anamnestic details, providing a comprehensive overview of each patient's medical history and relevant background factors related to our study. The database was saved in an Excel document. The statistics were run using IBM SPSS Statistics for Windows, Version 17 (Released 2008; IBM Corp., Armonk, New York, United States) and Microsoft Excel (Microsoft Corporation, Redmond, USA). In the first part of our analysis, we described the entire database using plots and frequency tables and calculated the central tendency and dispersion parameters. Next, we tested the data distribution by applying the Kolmogorov-Smirnov test. We used the Mann-Whitney and ANOVA one-way tests for the statistical analyses. The Mann-Whitney test was used to compare two independent groups when the data was not normally distributed, while the ANOVA test was used to compare the means of three or more groups. At the end of our study, we applied a correlation analysis to see if there was an association between the IL-6 and the CRP values. For all the statistical analyses, we considered α = 0.05 as the confidence level.

Ethical approval was waived for this study due to its retrospective design, which involved the analysis of existing data from medical records without direct patient intervention or identification. All data were anonymized to protect patient confidentiality.

## Results

The database contains 200 patients treated in our hospital during a one-year period. We have collected information about the mother’s age, the gestational period, the newborn's weight, the APGARE score, the IL-6, and CRP values if there are infections or acute diseases if the patient had hypertension, thrombophilia, hypothyroidism, diabetes, rupture of membranes, or if the mother is a smoker. In the first part, we made frequency tables for the qualitative variables, and we calculated the central tendency and dispersion parameters for the numerical variables. Of the 39 patients (9.5%) who had infections, 10 patients (5%) had fatal maternal infections, 15 patients (7.5%) had urinary infections, and 14 patients (7%) had candida infection. Of the 19 patients (9.5%) who had acute diseases, 12 patients (6%) had previa placenta, and seven patients (3.5%) had intrapartum hemorrhage. Of the 200 patients/200 newborns, 107 babies (53.5%) came from premature birth (the gestational period of less than 37 weeks). All the results are presented in Table 1 and Table 2.

**Table 1:** Data frequency We calculated the frequency table for the infections, acute diseases, hypertension, thrombophilia, hypothyroidism, diabetes, broken membranes, and smoker parameters.

Variable	Number		Percentage	
	Yes	No	% Yes	% No
Infections	39	161	19.5%	80.5%
Acute Diseases	19	181	9.5%	90.5%
Hypertension	41	159	20.5%	79.5%
Thrombophilia	42	158	21%	79%
Hypothyroidism	27	173	13.5	86.5%
Diabetes	31	169	15.5%	84.5%
Broken Membranes	71	129	35.5%	64.5%
Smoker	87	113	43.5%	56.5%

**Table 2:** Variables and statistical parameters The table shows the calculated central tendency and dispersion parameters for the numerical variables from our study.

Statistics	Mother's Age	Gestational Period	Newborn Weight	APGAR	IL-6	CRP
Mean	29.36	34.88	2606.10	8.13	29.64	9.92
Standard Error	0.40	0.25	59.58	0.10	2.45	0.41
Median	29	33.5	2390	8	13.7	8.37
Mode	32	38	1800	9	1.99	14.44
Standard Deviation	5.61	3.56	842.58	1.39	34.62	5.74
Sample Variance	31.42	12.65	709947.28	1.93	1198.20	32.94
Kurtosis	-0.61	-1.50	-1.31	4.23	0.71	-0.21
Skewness	-0.28	-0.10	0.21	-1.42	1.31	0.97
Range	23	11	3460	9	144.7	19.65
Minimum	16	29	950	1	0.7	3.93
Maximum	39	40	4410	10	145.4	23.58
Sum	5871	6975	521220	1625	5927.37	1984.73
Count	200	200	200	200	200	200

**Figure 1:**
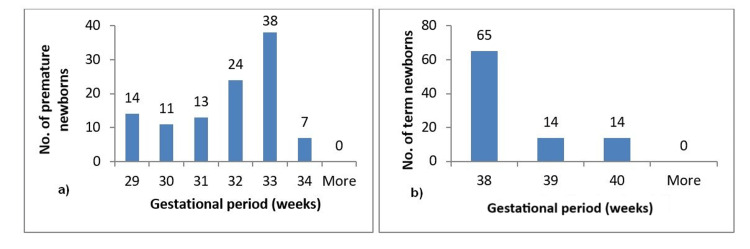
Distribution of premature and term births across gestational ages a and b represent the distribution of premature and term births across gestational ages using histograms

**Figure 2:**
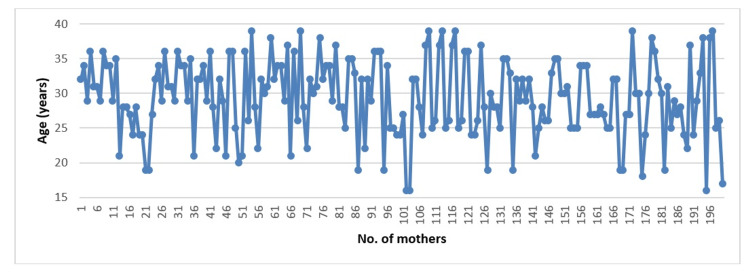
Mother’s age distribution Mother's age distribution is shown using a line chart.

**Figure 3:**
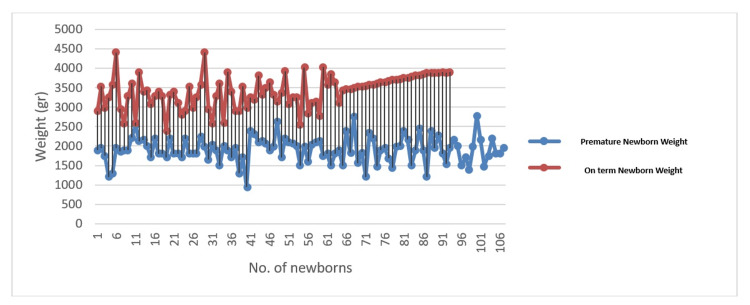
Newborn weight distribution The figure represents the weight distribution in newborns separated into two subgroups: premature babies (less than 37 pregnancy weeks) and on term babies (more than 37 pregnancy weeks)

For the most significant results, we plotted the data in order to have a better representation (Figures 1-3).

Furthermore, we tested the data distribution by applying the Kolmogorov-Smirnov test, and we obtained p-values less than 0.05, which can be interpreted as indicating that the data are not normally distributed. Therefore, we will use non-parametric tests in our statistics.

We tested to see if the IL-6 and CRP values were sensitive to infection. For this, we applied a one-way ANOVA test, and we obtained significant differences. The IL-6 and CRP values are the most increased in a candida infection. When we considered the infection variable as a dichotomous variable (infection present/ absent) and applied the Mann-Whitney test, we obtained extremely significant differences in the IL-6 and CRP values. Also, we tested to see if the smoking habit can influence the IL-6 and CRP values. We applied the Mann-Whitney test again, but in this case, we obtained insignificant differences. Thus, we can say that the infection can influence significantly the IL-6 and CRP values, but these values are not sensitive to the smoking habit.

Results indicated that the area under the curve (AUC) for IL-6 and CRP was 0.87 and 0.80, respectively. The optimal IL-6 cutoff was 181 pg/ml, yielding a sensitivity of 80.1%, a specificity of 85.7%, a positive predictive value (PPV) of 84.6%, and a negative predictive value (NPV) of 81.8%. For CRP, the cutoff was 3.78 mg/dl, with a sensitivity of 61%, a specificity of 90.5%, a PPV of 86.2%, and an NPV of 70.3%. Among culture-positive cases, IL-6 and CRP sensitivities were 90% and 80%, respectively, while in culture-negative cases, sensitivities were 71.4% for IL-6 and 42.8% for CRP. In EOS, IL-6 and CRP sensitivities were 86.3% and 50%, respectively, whereas in LOS, IL-6 and CRP sensitivities were 73.6% and 72.6%, respectively.

**Figure 4:**
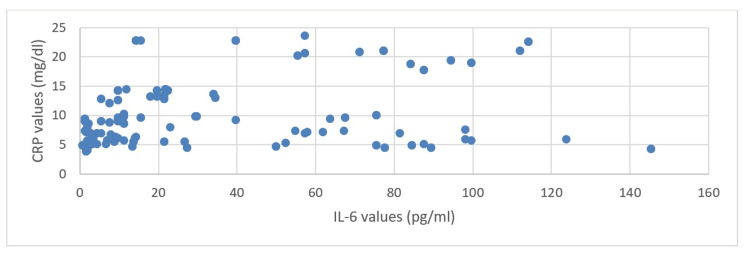
The association between the IL-6 and CRP values IL-6: Interleukin-6; CRP: C-reactive protein

At the end of our study, we ran a correlation analysis in order to see if there is an association between the IL-6 and CRP values. By applying a correlation model, we obtained a significant, positive correlation (Figure 4).

## Discussion

Neonatal complications should be analyzed from a complex and mutually interactional perspective between the mother and her neonate. On the one hand, there might be a biological substrate in determining inflammation processes in neonates vertically transmitted from the mother. One study found a significant positive correlation between the total antioxidant capacity level and irisin concentration in newborns. In both study groups, maternal and neonatal levels of IL-1β, IL-1RA, IL-5, IL-7, and interferon gamma-induced protein (IP)-10 showed a significant positive correlation with irisin concentrations [14]. It is noteworthy to mention that even certain psychopathological conditions in mothers, such as peripartum depression and peripartum anxiety, have been revealed to be associated with increased serum levels of Interleukin-6, TNF- α, and IL-8 [15,16]. 

Our study results showed that both IL-6 and CRP are valuable biomarkers for predicting NS, with IL-6 showing stronger overall performance. The AUC values of 0.87 for IL-6 and 0.80 for CRP indicate that IL-6 has a slightly higher discriminatory ability in identifying NS compared to CRP. 

For IL-6, the optimal cutoff value of 181 pg/ml demonstrated a high sensitivity of 80.1% and specificity of 85.7%, meaning it is fairly accurate in correctly identifying both affected and unaffected infants. The PPV (84.6%) and NPV (81.8%) further suggest that IL-6 is reliable in predicting both positive and negative cases of sepsis. CRP, on the other hand, had a lower sensitivity of 61% but a higher specificity of 90.5%, indicating that it is more effective at ruling out non-sepsis cases. The PPV of 86.2% suggests that CRP is quite reliable in confirming positive cases, while its NPV of 70.3% reflects a somewhat lower capacity to exclude negative cases compared to IL-6.

In culture-positive cases, both biomarkers performed well, with IL-6 having a higher sensitivity (90%) than CRP (80%). However, in culture-negative cases, IL-6 outperformed CRP, with a sensitivity of 71.4% compared to CRP's 42.8%, showing IL-6's stronger predictive value in these cases. Regarding EOS, IL-6 exhibited a high sensitivity (86.3%) compared to CRP (50%), while in LOS, both biomarkers demonstrated similar sensitivity (IL-6 at 73.6% and CRP at 72.6%). This suggests that IL-6 is particularly useful in detecting EOS, while both IL-6 and CRP are relatively comparable in diagnosing LOS.

Overall, the results showed that IL-6 is a more reliable indicator of newborn sepsis, showing improved performance and sensitivity in both EOS and culture-negative cases. Because of its high specificity, CRP is still a helpful adjuvant, particularly when ruling out sepsis.

Although our investigation generated significant findings, it was not without limitations. While 200 participants are a suitable sample size for many statistical analyses, it might not accurately reflect the diversity of the general population, which could restrict the findings' applicability to other demographic groups or healthcare environments. Additionally, our results may be limited due to the single-center nature of the study. Multi-center randomized studies are required to clearly determine the optimal detection approach because of the pathology's severity and potential complications.

Procalcitonin (PCT) is another important marker for NS. It is an essential biomarker in diagnosing and managing NS due to its quick response to bacterial infection and its specificity for bacterial origins over viral or inflammatory conditions [17].

On the other hand, the presence of pregnancy and neonatal complications may be considered psychosocial stressors, especially in younger mothers, that may trigger a mood or anxiety episode in women who are at increased risk. In this case, psychogenic mechanisms rather than biological substrates are presumed to underly the clinical manifestations. Moreover, psychosocial stress has been found to activate macrophages through the sympathetic chain. Therefore, activated macrophages lead to the release of inflammatory markers in circulation, such as pro-inflammatory cytokines, chemokines, adhesion molecules, and acute-phase reactants. Besides the inflammatory role, cytokines also have a signaling role promoting neuroinflammation and activation of microglia and astrocytes. These actions may contribute to neuronal damage underlying different psychopathological conditions [18].

Antenatal depression and anxiety are associated with an increased level of cortisol caused by the pre-existence of hypothalamic-pituitary-adrenal anomalies in mothers [19]. One study has evidenced that in women with major psychiatric disorders, stress-related neuroendocrine responses are diminished, accompanied by increased immune activation and reduced sensitivity to glucocorticoids [20]. Furthermore, other results indicate that hypercortisolemia is linked to transient depressive states, while hypocortisolemia is related to chronic postpartum depression [21].

Among the risk factors during pregnancy for NS are maternal infections, preterm birth, prolonged rupture of membranes, preeclampsia and other hypertensive disorders, invasive procedures during pregnancy or delivery, maternal pathology, multiple pregnancies, maternal obesity, poor prenatal care, inadequate maternal nutrition, and vitamin deficiencies.

Pregnancy complications such as preeclampsia, gestational hypertension, and inflammation have an important impact on arterial stiffness in pregnant women with multiple effects, possibly also an increased risk for NS [22]. Further research is needed to explore this correlation and determine whether improving maternal vascular health can help reduce neonatal infections, but a personalized approach to preventing vascular modification during pregnancy is recommended [23].

Maternal malnutrition and deficiencies in crucial nutrients like vitamin D and iron can weaken the immune system of both the mother and fetus. Studies suggest that maternal vitamin D deficiency is associated with an increased risk of preterm birth and infections, both of which are risk factors for NS [24].

## Conclusions

Infections significantly alter the levels of IL-6 and CRP in the body, proving that both biomarkers can indicate an inflammatory response due to infection, but they remain unaffected by smoking habits. There is a notable correlation between IL-6 and CRP levels; in particular, elevated IL-6 levels are likely to correspond to increased CRP values.

Although CRP and PCT are commonly used in laboratory testing, they are insufficient on their own for the early diagnosis of sepsis. IL-6 emerges as a more reliable biomarker for the early detection of sepsis due to its high sensitivity and good specificity. Its diagnostic value surpasses that of CRP, suggesting that IL-6 testing should be prioritized in clinical evaluations for suspected sepsis to improve early diagnosis and treatment strategies.
